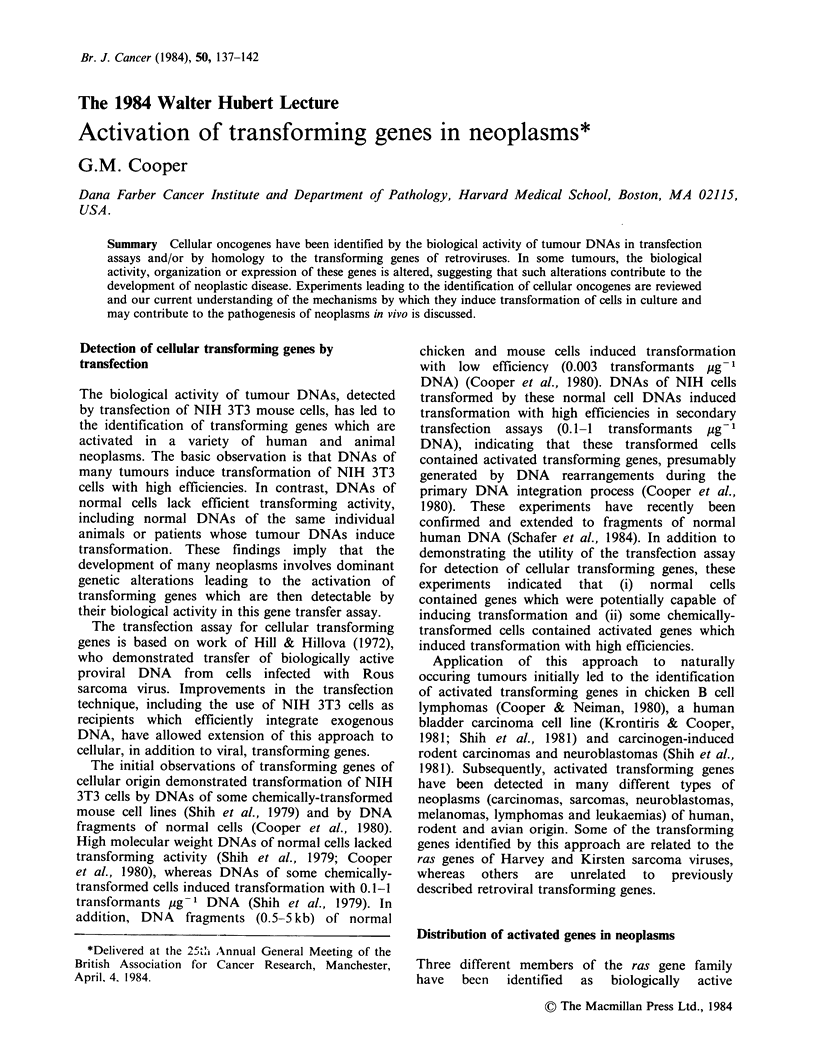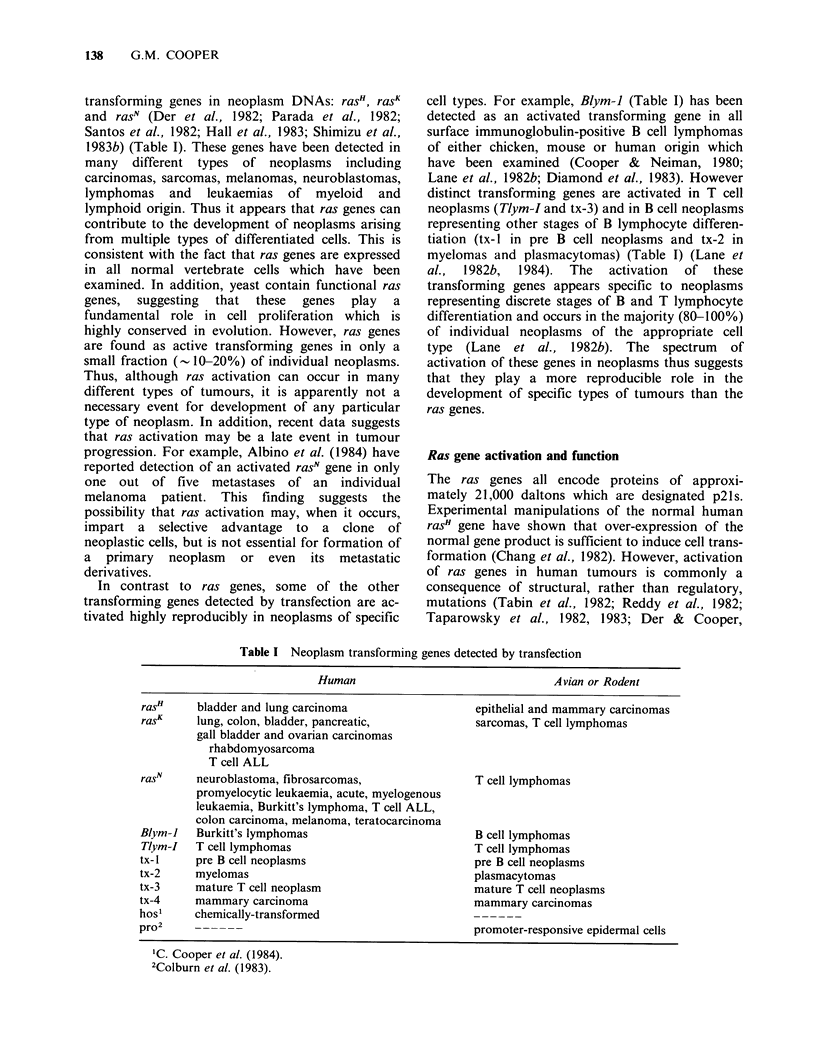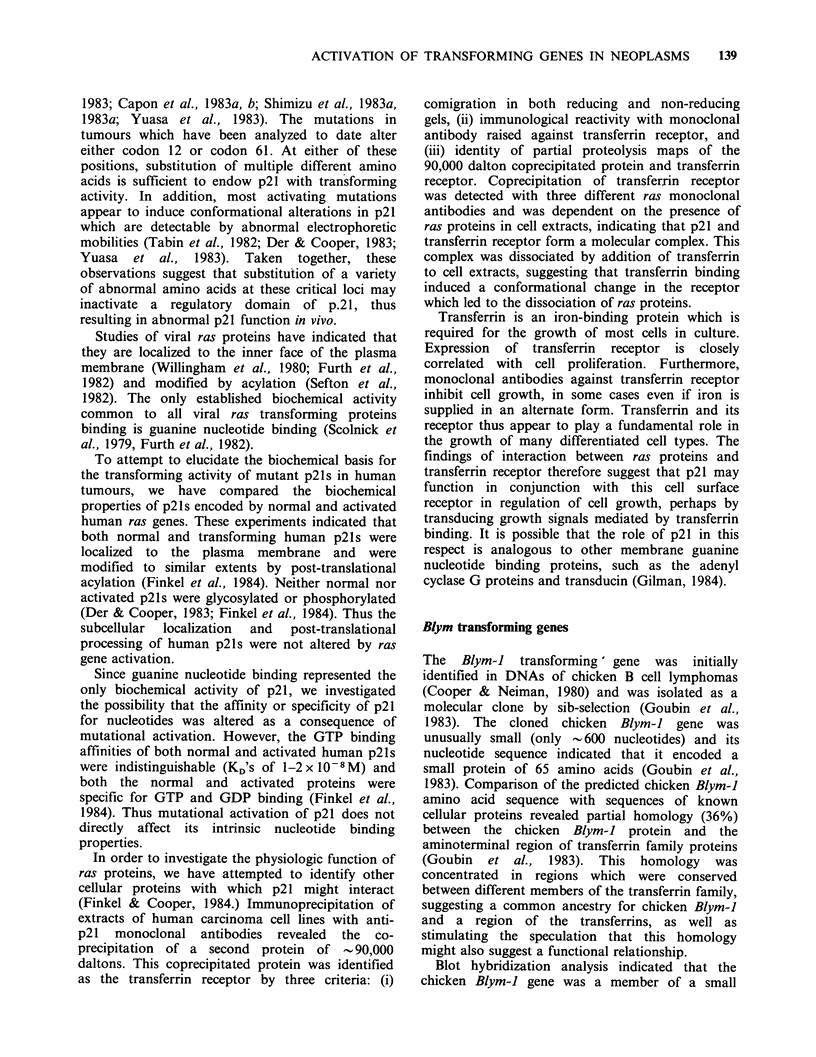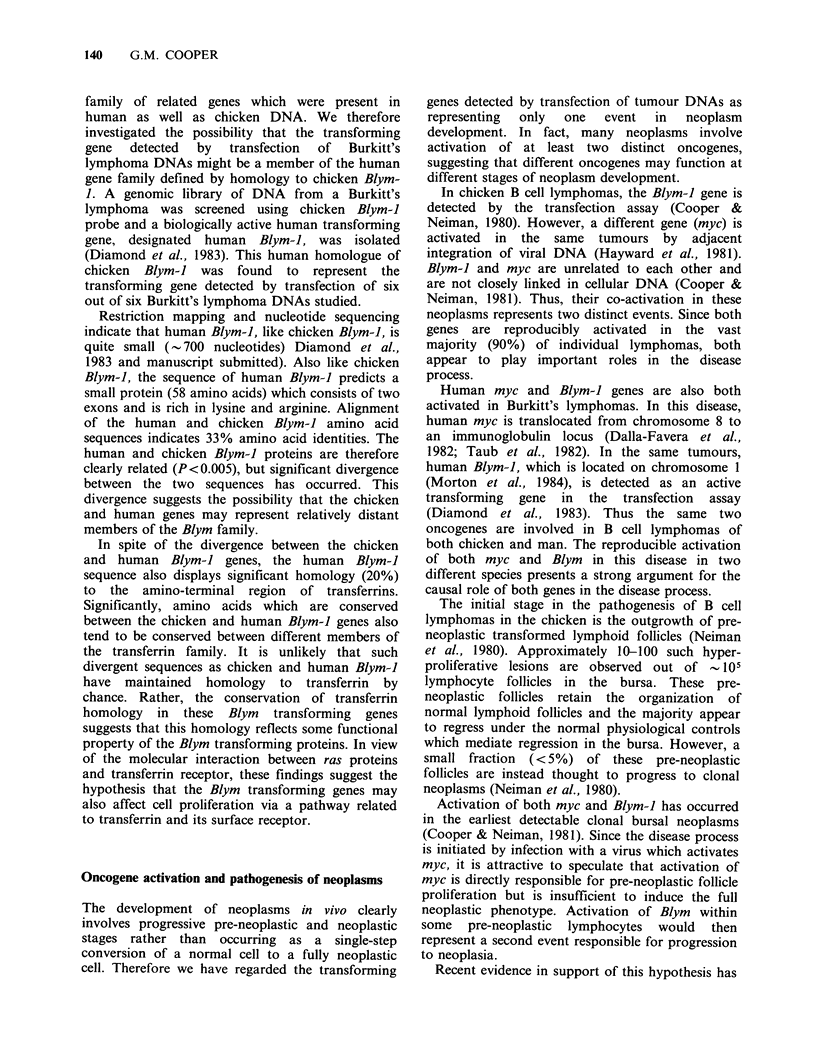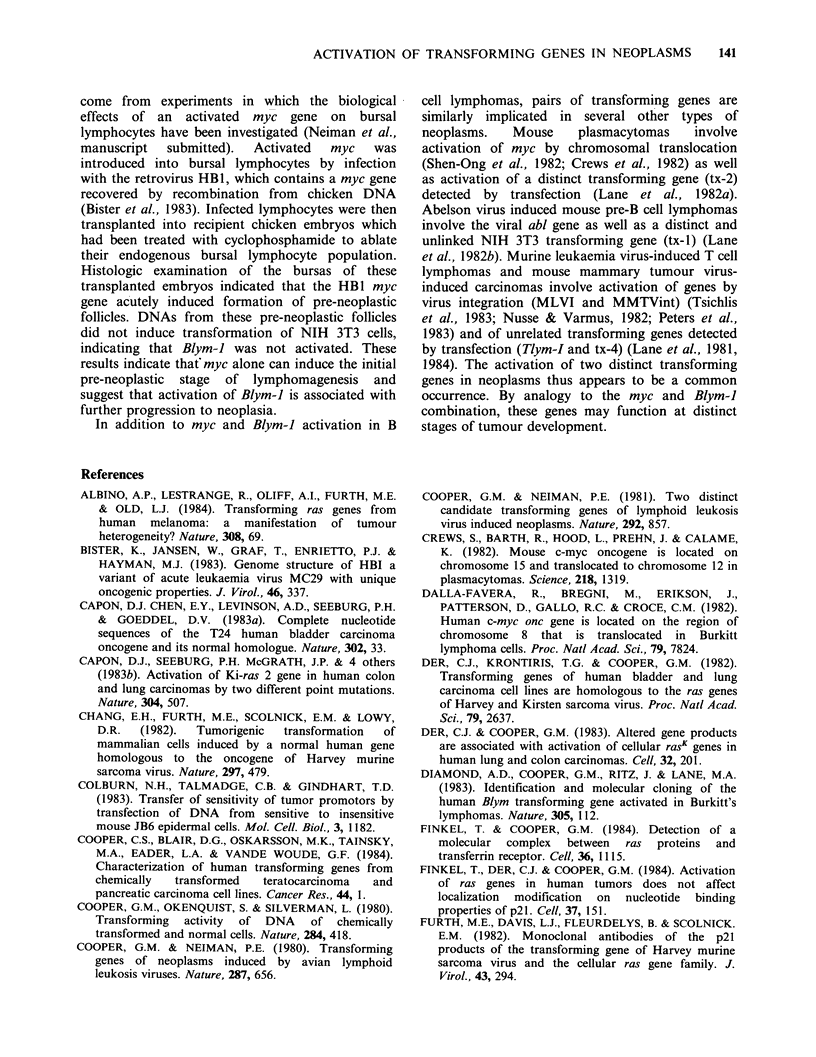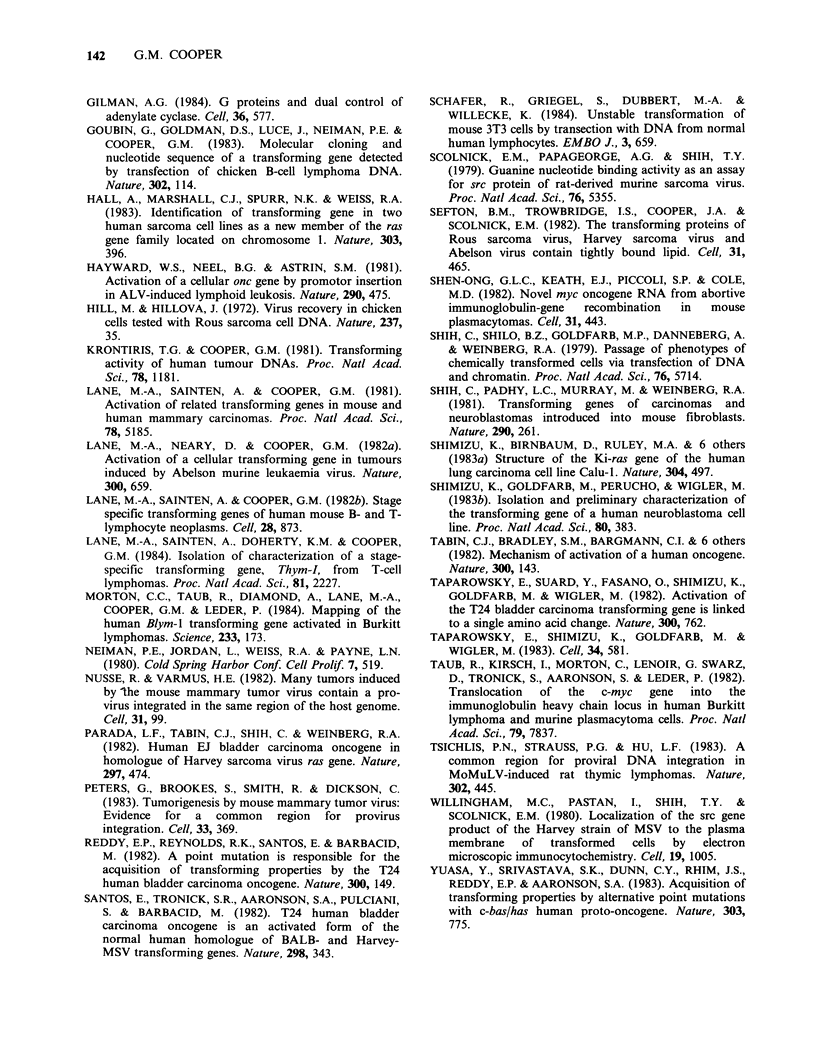# The 1984 Walter Hubert lecture. Activation of transforming genes in neoplasms.

**DOI:** 10.1038/bjc.1984.155

**Published:** 1984-08

**Authors:** G. M. Cooper

## Abstract

Cellular oncogenes have been identified by the biological activity of tumour DNAs in transfection assays and/or by homology to the transforming genes of retroviruses. In some tumours, the biological activity, organization or expression of these genes is altered, suggesting that such alterations contribute to the development of neoplastic disease. Experiments leading to the identification of cellular oncogenes are reviewed and our current understanding of the mechanisms by which they induce transformation of cells in culture and may contribute to the pathogenesis of neoplasms in vivo is discussed.


*Br. J. Cancer* (1984), **50**, 137–142

### The 1984 Walter Hubert Lecture

### Activation of transforming genes in neoplasms*

G.M. Cooper

*Dana Farber Cancer Institute and Department of Pathology, Harvard Medical School, Boston, MA 02115, USA.*

**Summary** Cellular oncogenes have been identified by the biological activity of tumour DNAs in transfection assays and/or by homology to the transforming genes of retroviruses. In some tumours, the biological activity, organization or expression of these genes is altered, suggesting that such alterations contribute to the development of neoplastic disease. Experiments leading to the identification of cellular oncogenes are reviewed and our current understanding of the mechanisms by which they induce transformation of cells in culture and may contribute to the pathogenesis of neoplasms *in vivo* is discussed.

### Detection of cellular transforming genes by transfection

The biological activity of tumour DNAs, detected by transfection of NIH 3T3 mouse cells, has led to the identification of transforming genes which are activated in a variety of human and animal neoplasms. The basic observation is that DNAs of many tumours induce transformation of NIH 3T3 cells with high efficiencies. In contrast, DNAs of normal cells lack efficient transforming activity, including normal DNAs of the same individual animals or patients whose tumour DNAs induce transformation. These findings imply that the development of many neoplasms involves dominant genetic alterations leading to the activation of transforming genes which are then detectable by their biological activity in this gene transfer assay.

The transfection assay for cellular transforming genes is based on work of Hill & Hillova (1972), who demonstrated transfer of biologically active proviral DNA from cells infected with Rous sarcoma virus. Improvements in the transfection technique, including the use of NIH 3T3 cells as recipients which efficiently integrate exogenous DNA, have allowed extension of this approach to cellular, in addition to viral, transforming genes.

The initial observations of transforming genes of cellular origin demonstrated transformation of NIH 3T3 cells by DNAs of some chemically-transformed mouse cell lines (Shih *et al.*, 1979) and by DNA fragments of normal cells (Cooper *et al.*, 1980). High molecular weight DNAs of normal cells lacked transforming activity (Shih *et al.*, 1979; Cooper *et al.*, 1980), whereas DNAs of some chemically-transformed cells induced transformation with 0.1–1 transformants $\mu g^{-1}$ DNA (Shih *et al.*, 1979). In addition, DNA fragments (0.5–5 kb) of normal chicken and mouse cells induced transformation with low efficiency (0.003 transformants $\mu g^{-1}$ DNA) (Cooper *et al.*, 1980). DNAs of NIH cells transformed by these normal cell DNAs induced transformation with high efficiencies in secondary transfection assays (0.1–1 transformants $\mu g^{-1}$ DNA), indicating that these transformed cells contained activated transforming genes, presumably generated by DNA rearrangements during the primary DNA integration process (Cooper *et al.*, 1980). These experiments have recently been confirmed and extended to fragments of normal human DNA (Schafer *et al.*, 1984). In addition to demonstrating the utility of the transfection assay for detection of cellular transforming genes, these experiments indicated that (i) normal cells contained genes which were potentially capable of inducing transformation and (ii) some chemically-transformed cells contained activated genes which induced transformation with high efficiencies.

Application of this approach to naturally occuring tumours initially led to the identification of activated transforming genes in chicken B cell lymphomas (Cooper & Neiman, 1980), a human bladder carcinoma cell line (Krontiris & Cooper, 1981; Shih *et al.*, 1981) and carcinogen-induced rodent carcinomas and neuroblastomas (Shih *et al.*, 1981). Subsequently, activated transforming genes have been detected in many different types of neoplasms (carcinomas, sarcomas, neuroblastomas, melanomas, lymphomas and leukaemias) of human, rodent and avian origin. Some of the transforming genes identified by this approach are related to the *ras* genes of Harvey and Kirsten sarcoma viruses, whereas others are unrelated to previously described retroviral transforming genes.

### Distribution of activated genes in neoplasms

Three different members of the *ras* gene family have been identified as biologically active

*Delivered at the 25th Annual General Meeting of the British Association for Cancer Research, Manchester, April, 4, 1984.

© The Macmillan Press Ltd., 1984

transforming genes in neoplasm DNAs: $ras^H$, $ras^K$ and $ras^N$ (Der *et al.*, 1982; Parada *et al.*, 1982; Santos *et al.*, 1982; Hall *et al.*, 1983; Shimizu *et al.*, 1983*b*) (Table I). These genes have been detected in many different types of neoplasms including carcinomas, sarcomas, melanomas, neuroblastomas, lymphomas and leukaemias of myeloid and lymphoid origin. Thus it appears that *ras* genes can contribute to the development of neoplasms arising from multiple types of differentiated cells. This is consistent with the fact that *ras* genes are expressed in all normal vertebrate cells which have been examined. In addition, yeast contain functional *ras* genes, suggesting that these genes play a fundamental role in cell proliferation which is highly conserved in evolution. However, *ras* genes are found as active transforming genes in only a small fraction (~10–20%) of individual neoplasms. Thus, although *ras* activation can occur in many different types of tumours, it is apparently not a necessary event for development of any particular type of neoplasm. In addition, recent data suggests that *ras* activation may be a late event in tumour progression. For example, Albino *et al.* (1984) have reported detection of an activated $ras^N$ gene in only one out of five metastases of an individual melanoma patient. This finding suggests the possibility that *ras* activation may, when it occurs, impart a selective advantage to a clone of neoplastic cells, but is not essential for formation of a primary neoplasm or even its metastatic derivatives.

In contrast to *ras* genes, some of the other transforming genes detected by transfection are activated highly reproducibly in neoplasms of specific cell types. For example, *Blym-1* (Table I) has been detected as an activated transforming gene in all surface immunoglobulin-positive B cell lymphomas of either chicken, mouse or human origin which have been examined (Cooper & Neiman, 1980; Lane *et al.*, 1982*b*; Diamond *et al.*, 1983). However distinct transforming genes are activated in T cell neoplasms (*Tlym-I* and tx-3) and in B cell neoplasms representing other stages of B lymphocyte differentiation (tx-1 in pre B cell neoplasms and tx-2 in myelomas and plasmacytomas) (Table I) (Lane *et al.*, 1982*b*, 1984). The activation of these transforming genes appears specific to neoplasms representing discrete stages of B and T lymphocyte differentiation and occurs in the majority (80–100%) of individual neoplasms of the appropriate cell type (Lane *et al.*, 1982*b*). The spectrum of activation of these genes in neoplasms thus suggests that they play a more reproducible role in the development of specific types of tumours than the *ras* genes.

### *Ras* gene activation and function

The *ras* genes all encode proteins of approximately 21,000 daltons which are designated p21s. Experimental manipulations of the normal human $ras^H$ gene have shown that over-expression of the normal gene product is sufficient to induce cell transformation (Chang *et al.*, 1982). However, activation of *ras* genes in human tumours is commonly a consequence of structural, rather than regulatory, mutations (Tabin *et al.*, 1982; Reddy *et al.*, 1982; Taparowsky *et al.*, 1982, 1983; Der & Cooper,

**Table I** Neoplasm transforming genes detected by transfection

| | *Human* | *Avian or Rodent* |
|---|---|---|
| $ras^H$ | bladder and lung carcinoma | epithelial and mammary carcinomas |
| $ras^K$ | lung, colon, bladder, pancreatic, gall bladder and ovarian carcinomas rhabdomyosarcoma T cell ALL | sarcomas, T cell lymphomas |
| $ras^N$ | neuroblastoma, fibrosarcomas, promyelocytic leukaemia, acute, myelogenous leukaemia, Burkitt's lymphoma, T cell ALL, colon carcinoma, melanoma, teratocarcinoma | T cell lymphomas |
| *Blym-1* | Burkitt's lymphomas | B cell lymphomas |
| *Tlym-I* | T cell lymphomas | T cell lymphomas |
| tx-1 | pre B cell neoplasms | pre B cell neoplasms |
| tx-2 | myelomas | plasmacytomas |
| tx-3 | mature T cell neoplasm | mature T cell neoplasms |
| tx-4 | mammary carcinoma | mammary carcinomas |
| hos[1] | chemically-transformed | —————— |
| pro[2] | —————— | promoter-responsive epidermal cells |

[1]C. Cooper *et al.* (1984).
[2]Colburn *et al.* (1983).

1983; Capon *et al.*, 1983*a, b*; Shimizu *et al.*, 1983*a*, 1983*a*; Yuasa *et al.*, 1983). The mutations in tumours which have been analyzed to date alter either codon 12 or codon 61. At either of these positions, substitution of multiple different amino acids is sufficient to endow p21 with transforming activity. In addition, most activating mutations appear to induce conformational alterations in p21 which are detectable by abnormal electrophoretic mobilities (Tabin *et al.*, 1982; Der & Cooper, 1983; Yuasa *et al.*, 1983). Taken together, these observations suggest that substitution of a variety of abnormal amino acids at these critical loci may inactivate a regulatory domain of p.21, thus resulting in abnormal p21 function *in vivo*.

Studies of viral *ras* proteins have indicated that they are localized to the inner face of the plasma membrane (Willingham *et al.*, 1980; Furth *et al.*, 1982) and modified by acylation (Sefton *et al.*, 1982). The only established biochemical activity common to all viral *ras* transforming proteins binding is guanine nucleotide binding (Scolnick *et al.*, 1979, Furth *et al.*, 1982).

To attempt to elucidate the biochemical basis for the transforming activity of mutant p21s in human tumours, we have compared the biochemical properties of p21s encoded by normal and activated human *ras* genes. These experiments indicated that both normal and transforming human p21s were localized to the plasma membrane and were modified to similar extents by post-translational acylation (Finkel *et al.*, 1984). Neither normal nor activated p21s were glycosylated or phosphorylated (Der & Cooper, 1983; Finkel *et al.*, 1984). Thus the subcellular localization and post-translational processing of human p21s were not altered by *ras* gene activation.

Since guanine nucleotide binding represented the only biochemical activity of p21, we investigated the possibility that the affinity or specificity of p21 for nucleotides was altered as a consequence of mutational activation. However, the GTP binding affinities of both normal and activated human p21s were indistinguishable ($K_D$'s of $1\text{–}2 \times 10^{-8}$M) and both the normal and activated proteins were specific for GTP and GDP binding (Finkel *et al.*, 1984). Thus mutational activation of p21 does not directly affect its intrinsic nucleotide binding properties.

In order to investigate the physiologic function of *ras* proteins, we have attempted to identify other cellular proteins with which p21 might interact (Finkel & Cooper, 1984.) Immunoprecipitation of extracts of human carcinoma cell lines with anti-p21 monoclonal antibodies revealed the co-precipitation of a second protein of ~90,000 daltons. This coprecipitated protein was identified as the transferrin receptor by three criteria: (i) comigration in both reducing and non-reducing gels, (ii) immunological reactivity with monoclonal antibody raised against transferrin receptor, and (iii) identity of partial proteolysis maps of the 90,000 dalton coprecipitated protein and transferrin receptor. Coprecipitation of transferrin receptor was detected with three different *ras* monoclonal antibodies and was dependent on the presence of *ras* proteins in cell extracts, indicating that p21 and transferrin receptor form a molecular complex. This complex was dissociated by addition of transferrin to cell extracts, suggesting that transferrin binding induced a conformational change in the receptor which led to the dissociation of *ras* proteins.

Transferrin is an iron-binding protein which is required for the growth of most cells in culture. Expression of transferrin receptor is closely correlated with cell proliferation. Furthermore, monoclonal antibodies against transferrin receptor inhibit cell growth, in some cases even if iron is supplied in an alternate form. Transferrin and its receptor thus appear to play a fundamental role in the growth of many differentiated cell types. The findings of interaction between *ras* proteins and transferrin receptor therefore suggest that p21 may function in conjunction with this cell surface receptor in regulation of cell growth, perhaps by transducing growth signals mediated by transferrin binding. It is possible that the role of p21 in this respect is analogous to other membrane guanine nucleotide binding proteins, such as the adenyl cyclase G proteins and transducin (Gilman, 1984).

### *Blym* transforming genes

The *Blym-1* transforming' gene was initially identified in DNAs of chicken B cell lymphomas (Cooper & Neiman, 1980) and was isolated as a molecular clone by sib-selection (Goubin *et al.*, 1983). The cloned chicken *Blym-1* gene was unusually small (only ~600 nucleotides) and its nucleotide sequence indicated that it encoded a small protein of 65 amino acids (Goubin *et al.*, 1983). Comparison of the predicted chicken *Blym-1* amino acid sequence with sequences of known cellular proteins revealed partial homology (36%) between the chicken *Blym-1* protein and the aminoterminal region of transferrin family proteins (Goubin *et al.*, 1983). This homology was concentrated in regions which were conserved between different members of the transferrin family, suggesting a common ancestry for chicken *Blym-1* and a region of the transferrins, as well as stimulating the speculation that this homology might also suggest a functional relationship.

Blot hybridization analysis indicated that the chicken *Blym-1* gene was a member of a small family of related genes which were present in human as well as chicken DNA. We therefore investigated the possibility that the transforming gene detected by transfection of Burkitt's lymphoma DNAs might be a member of the human gene family defined by homology to chicken *Blym-1*. A genomic library of DNA from a Burkitt's lymphoma was screened using chicken *Blym-1* probe and a biologically active human transforming gene, designated human *Blym-1*, was isolated (Diamond *et al.*, 1983). This human homologue of chicken *Blym-1* was found to represent the transforming gene detected by transfection of six out of six Burkitt's lymphoma DNAs studied.

Restriction mapping and nucleotide sequencing indicate that human *Blym-1*, like chicken *Blym-1*, is quite small (~700 nucleotides) Diamond *et al.*, 1983 and manuscript submitted). Also like chicken *Blym-1*, the sequence of human *Blym-1* predicts a small protein (58 amino acids) which consists of two exons and is rich in lysine and arginine. Alignment of the human and chicken *Blym-1* amino acid sequences indicates 33% amino acid identities. The human and chicken *Blym-1* proteins are therefore clearly related ($P<0.005$), but significant divergence between the two sequences has occurred. This divergence suggests the possibility that the chicken and human genes may represent relatively distant members of the *Blym* family.

In spite of the divergence between the chicken and human *Blym-1* genes, the human *Blym-1* sequence also displays significant homology (20%) to the amino-terminal region of transferrins. Significantly, amino acids which are conserved between the chicken and human *Blym-1* genes also tend to be conserved between different members of the transferrin family. It is unlikely that such divergent sequences as chicken and human *Blym-1* have maintained homology to transferrin by chance. Rather, the conservation of transferrin homology in these *Blym* transforming genes suggests that this homology reflects some functional property of the *Blym* transforming proteins. In view of the molecular interaction between *ras* proteins and transferrin receptor, these findings suggest the hypothesis that the *Blym* transforming genes may also affect cell proliferation via a pathway related to transferrin and its surface receptor.

### Oncogene activation and pathogenesis of neoplasms

The development of neoplasms *in vivo* clearly involves progressive pre-neoplastic and neoplastic stages rather than occurring as a single-step conversion of a normal cell to a fully neoplastic cell. Therefore we have regarded the transforming genes detected by transfection of tumour DNAs as representing only one event in neoplasm development. In fact, many neoplasms involve activation of at least two distinct oncogenes, suggesting that different oncogenes may function at different stages of neoplasm development.

In chicken B cell lymphomas, the *Blym-1* gene is detected by the transfection assay (Cooper & Neiman, 1980). However, a different gene (*myc*) is activated in the same tumours by adjacent integration of viral DNA (Hayward *et al.*, 1981). *Blym-1* and *myc* are unrelated to each other and are not closely linked in cellular DNA (Cooper & Neiman, 1981). Thus, their co-activation in these neoplasms represents two distinct events. Since both genes are reproducibly activated in the vast majority (90%) of individual lymphomas, both appear to play important roles in the disease process.

Human *myc* and *Blym-1* genes are also both activated in Burkitt's lymphomas. In this disease, human *myc* is translocated from chromosome 8 to an immunoglobulin locus (Dalla-Favera *et al.*, 1982; Taub *et al.*, 1982). In the same tumours, human *Blym-1*, which is located on chromosome 1 (Morton *et al.*, 1984), is detected as an active transforming gene in the transfection assay (Diamond *et al.*, 1983). Thus the same two oncogenes are involved in B cell lymphomas of both chicken and man. The reproducible activation of both *myc* and *Blym* in this disease in two different species presents a strong argument for the causal role of both genes in the disease process.

The initial stage in the pathogenesis of B cell lymphomas in the chicken is the outgrowth of pre-neoplastic transformed lymphoid follicles (Neiman *et al.*, 1980). Approximately 10–100 such hyper-proliferative lesions are observed out of $\sim 10^5$ lymphocyte follicles in the bursa. These pre-neoplastic follicles retain the organization of normal lymphoid follicles and the majority appear to regress under the normal physiological controls which mediate regression in the bursa. However, a small fraction (<5%) of these pre-neoplastic follicles are instead thought to progress to clonal neoplasms (Neiman *et al.*, 1980).

Activation of both *myc* and *Blym-1* has occurred in the earliest detectable clonal bursal neoplasms (Cooper & Neiman, 1981). Since the disease process is initiated by infection with a virus which activates *myc*, it is attractive to speculate that activation of *myc* is directly responsible for pre-neoplastic follicle proliferation but is insufficient to induce the full neoplastic phenotype. Activation of *Blym* within some pre-neoplastic lymphocytes would then represent a second event responsible for progression to neoplasia.

Recent evidence in support of this hypothesis has come from experiments in which the biological effects of an activated *myc* gene on bursal lymphocytes have been investigated (Neiman *et al.*, manuscript submitted). Activated *myc* was introduced into bursal lymphocytes by infection with the retrovirus HB1, which contains a *myc* gene recovered by recombination from chicken DNA (Bister *et al.*, 1983). Infected lymphocytes were then transplanted into recipient chicken embryos which had been treated with cyclophosphamide to ablate their endogenous bursal lymphocyte population. Histologic examination of the bursas of these transplanted embryos indicated that the HB1 *myc* gene acutely induced formation of pre-neoplastic follicles. DNAs from these pre-neoplastic follicles did not induce transformation of NIH 3T3 cells, indicating that *Blym-1* was not activated. These results indicate that *myc* alone can induce the initial pre-neoplastic stage of lymphomagenesis and suggest that activation of *Blym-1* is associated with further progression to neoplasia.

In addition to *myc* and *Blym-1* activation in B cell lymphomas, pairs of transforming genes are similarly implicated in several other types of neoplasms. Mouse plasmacytomas involve activation of *myc* by chromosomal translocation (Shen-Ong *et al.*, 1982; Crews *et al.*, 1982) as well as activation of a distinct transforming gene (tx-2) detected by transfection (Lane *et al.*, 1982*a*). Abelson virus induced mouse pre-B cell lymphomas involve the viral *abl* gene as well as a distinct and unlinked NIH 3T3 transforming gene (tx-1) (Lane *et al.*, 1982*b*). Murine leukaemia virus-induced T cell lymphomas and mouse mammary tumour virus-induced carcinomas involve activation of genes by virus integration (MLVI and MMTVint) (Tsichlis *et al.*, 1983; Nusse & Varmus, 1982; Peters *et al.*, 1983) and of unrelated transforming genes detected by transfection (*Tlym-I* and tx-4) (Lane *et al.*, 1981, 1984). The activation of two distinct transforming genes in neoplasms thus appears to be a common occurrence. By analogy to the *myc* and *Blym-1* combination, these genes may function at distinct stages of tumour development.

### References

ALBINO, A.P., LESTRANGE, R., OLIFF, A.I., FURTH, M.E. & OLD, L.J. (1984). Transforming *ras* genes from human melanoma: a manifestation of tumour heterogeneity? *Nature*, **308**, 69.

BISTER, K., JANSEN, W., GRAF, T., ENRIETTO, P.J. & HAYMAN, M.J. (1983). Genome structure of HBI a variant of acute leukaemia virus MC29 with unique oncogenic properties. *J. Virol.*, **46**, 337.

CAPON, D.J. CHEN, E.Y., LEVINSON, A.D., SEEBURG, P.H. & GOEDDEL, D.V. (1983*a*). Complete nucleotide sequences of the T24 human bladder carcinoma oncogene and its normal homologue. *Nature*, **302**, 33.

CAPON, D.J., SEEBURG, P.H. McGRATH, J.P. & 4 others (1983*b*). Activation of Ki-*ras* 2 gene in human colon and lung carcinomas by two different point mutations. *Nature*, **304**, 507.

CHANG, E.H., FURTH, M.E., SCOLNICK, E.M. & LOWY, D.R. (1982). Tumorigenic transformation of mammalian cells induced by a normal human gene homologous to the oncogene of Harvey murine sarcoma virus. *Nature*, **297**, 479.

COLBURN, N.H., TALMADGE, C.B. & GINDHART, T.D. (1983). Transfer of sensitivity of tumor promotors by transfection of DNA from sensitive to insensitive mouse JB6 epidermal cells. *Mol. Cell. Biol.*, **3**, 1182.

COOPER, C.S., BLAIR, D.G., OSKARSSON, M.K., TAINSKY, M.A., EADER, L.A. & VANDE WOUDE, G.F. (1984). Characterization of human transforming genes from chemically transformed teratocarcinoma and pancreatic carcinoma cell lines. *Cancer Res.*, **44**, 1.

COOPER, G.M., OKENQUIST, S. & SILVERMAN, L. (1980). Transforming activity of DNA of chemically transformed and normal cells. *Nature*, **284**, 418.

COOPER, G.M. & NEIMAN, P.E. (1980). Transforming genes of neoplasms induced by avian lymphoid leukosis viruses. *Nature*, **287**, 656.

COOPER, G.M. & NEIMAN, P.E. (1981). Two distinct candidate transforming genes of lymphoid leukosis virus induced neoplasms. *Nature*, **292**, 857.

CREWS, S., BARTH, R., HOOD, L., PREHN, J. & CALAME, K. (1982). Mouse c-myc oncogene is located on chromosome 15 and translocated to chromosome 12 in plasmacytomas. *Science*, **218**, 1319.

DALLA-FAVERA, R., BREGNI, M., ERIKSON, J., PATTERSON, D., GALLO, R.C. & CROCE, C.M. (1982). Human c-*myc onc* gene is located on the region of chromosome 8 that is translocated in Burkitt lymphoma cells. *Proc. Natl Acad. Sci.*, **79**, 7824.

DER, C.J., KRONTIRIS, T.G. & COOPER, G.M. (1982). Transforming genes of human bladder and lung carcinoma cell lines are homologous to the *ras* genes of Harvey and Kirsten sarcoma virus. *Proc. Natl Acad. Sci.*, **79**, 2637.

DER, C.J. & COOPER, G.M. (1983). Altered gene products are associated with activation of cellular $ras^K$ genes in human lung and colon carcinomas. *Cell*, **32**, 201.

DIAMOND, A.D., COOPER, G.M., RITZ, J. & LANE, M.A. (1983). Identification and molecular cloning of the human *Blym* transforming gene activated in Burkitt's lymphomas. *Nature*, **305**, 112.

FINKEL, T. & COOPER, G.M. (1984). Detection of a molecular complex between *ras* proteins and transferrin receptor. *Cell*, **36**, 1115.

FINKEL, T., DER, C.J. & COOPER, G.M. (1984). Activation of *ras* genes in human tumors does not affect localization modification on nucleotide binding properties of p21. *Cell*, **37**, 151.

FURTH, M.E., DAVIS, L.J., FLEURDELYS, B. & SCOLNICK. E.M. (1982). Monoclonal antibodies of the p21 products of the transforming gene of Harvey murine sarcoma virus and the cellular *ras* gene family. *J. Virol.*, **43**, 294.

GILMAN, A.G. (1984). G proteins and dual control of adenylate cyclase. *Cell*, **36**, 577.

GOUBIN, G., GOLDMAN, D.S., LUCE, J., NEIMAN, P.E. & COOPER, G.M. (1983). Molecular cloning and nucleotide sequence of a transforming gene detected by transfection of chicken B-cell lymphoma DNA. *Nature*, **302**, 114.

HALL, A., MARSHALL, C.J., SPURR, N.K. & WEISS, R.A. (1983). Identification of transforming gene in two human sarcoma cell lines as a new member of the *ras* gene family located on chromosome 1. *Nature*, **303**, 396.

HAYWARD, W.S., NEEL, B.G. & ASTRIN, S.M. (1981). Activation of a cellular *onc* gene by promotor insertion in ALV-induced lymphoid leukosis. *Nature*, **290**, 475.

HILL, M. & HILLOVA, J. (1972). Virus recovery in chicken cells tested with Rous sarcoma cell DNA. *Nature*, **237**, 35.

KRONTIRIS, T.G. & COOPER, G.M. (1981). Transforming activity of human tumour DNAs. *Proc. Natl Acad. Sci.*, **78**, 1181.

LANE, M.-A., SAINTEN, A. & COOPER, G.M. (1981). Activation of related transforming genes in mouse and human mammary carcinomas. *Proc. Natl Acad. Sci.*, **78**, 5185.

LANE, M.-A., NEARY, D. & COOPER, G.M. (1982*a*). Activation of a cellular transforming gene in tumours induced by Abelson murine leukaemia virus. *Nature*, **300**, 659.

LANE, M.-A., SAINTEN, A. & COOPER, G.M. (1982*b*). Stage specific transforming genes of human mouse B- and T-lymphocyte neoplasms. *Cell*, **28**, 873.

LANE, M.-A., SAINTEN, A., DOHERTY, K.M. & COOPER, G.M. (1984). Isolation of characterization of a stage-specific transforming gene, *Thym-I*, from T-cell lymphomas. *Proc. Natl Acad. Sci.*, **81**, 2227.

MORTON, C.C., TAUB, R., DIAMOND, A., LANE, M.-A., COOPER, G.M. & LEDER, P. (1984). Mapping of the human *Blym*-1 transforming gene activated in Burkitt lymphomas. *Science*, **233**, 173.

NEIMAN, P.E., JORDAN, L., WEISS, R.A. & PAYNE, L.N. (1980). *Cold Spring Harbor Conf. Cell Prolif.* **7**, 519.

NUSSE, R. & VARMUS, H.E. (1982). Many tumors induced by the mouse mammary tumor virus contain a provirus integrated in the same region of the host genome. *Cell*, **31**, 99.

PARADA, L.F., TABIN, C.J., SHIH, C. & WEINBERG, R.A. (1982). Human EJ bladder carcinoma oncogene in homologue of Harvey sarcoma virus *ras* gene. *Nature*, **297**, 474.

PETERS, G., BROOKES, S., SMITH, R. & DICKSON, C. (1983). Tumorigenesis by mouse mammary tumor virus: Evidence for a common region for provirus integration. *Cell*, **33**, 369.

REDDY, E.P., REYNOLDS, R.K., SANTOS, E. & BARBACID, M. (1982). A point mutation is responsible for the acquisition of transforming properties by the T24 human bladder carcinoma oncogene. *Nature*, **300**, 149.

SANTOS, E., TRONICK, S.R., AARONSON, S.A., PULCIANI, S. & BARBACID, M. (1982). T24 human bladder carcinoma oncogene is an activated form of the normal human homologue of BALB- and Harvey-MSV transforming genes. *Nature*, **298**, 343.

SCHAFER, R., GRIEGEL, S., DUBBERT, M.-A. & WILLECKE, K. (1984). Unstable transformation of mouse 3T3 cells by transection with DNA from normal human lymphocytes. *EMBO J.*, **3**, 659.

SCOLNICK, E.M., PAPAGEORGE, A.G. & SHIH, T.Y. (1979). Guanine nucleotide binding activity as an assay for *src* protein of rat-derived murine sarcoma virus. *Proc. Natl Acad. Sci.*, **76**, 5355.

SEFTON, B.M., TROWBRIDGE, I.S., COOPER, J.A. & SCOLNICK, E.M. (1982). The transforming proteins of Rous sarcoma virus, Harvey sarcoma virus and Abelson virus contain tightly bound lipid. *Cell*, **31**, 465.

SHEN-ONG, G.L.C., KEATH, E.J., PICCOLI, S.P. & COLE, M.D. (1982). Novel *myc* oncogene RNA from abortive immunoglobulin-gene recombination in mouse plasmacytomas. *Cell*, **31**, 443.

SHIH, C., SHILO, B.Z., GOLDFARB, M.P., DANNEBERG, A. & WEINBERG, R.A. (1979). Passage of phenotypes of chemically transformed cells via transfection of DNA and chromatin. *Proc. Natl Acad. Sci.*, **76**, 5714.

SHIH, C., PADHY, L.C., MURRAY, M. & WEINBERG, R.A. (1981). Transforming genes of carcinomas and neuroblastomas introduced into mouse fibroblasts. *Nature*, **290**, 261.

SHIMIZU, K., BIRNBAUM, D., RULEY, M.A. & 6 others (1983*a*) Structure of the Ki-*ras* gene of the human lung carcinoma cell line Calu-1. *Nature*, **304**, 497.

SHIMIZU, K., GOLDFARB, M., PERUCHO, & WIGLER, M. (1983*b*). Isolation and preliminary characterization of the transforming gene of a human neuroblastoma cell line. *Proc. Natl Acad. Sci.*, **80**, 383.

TABIN, C.J., BRADLEY, S.M., BARGMANN, C.I. & 6 others (1982). Mechanism of activation of a human oncogene. *Nature*, **300**, 143.

TAPAROWSKY, E., SUARD, Y., FASANO, O., SHIMIZU, K., GOLDFARB, M. & WIGLER, M. (1982). Activation of the T24 bladder carcinoma transforming gene is linked to a single amino acid change. *Nature*, **300**, 762.

TAPAROWSKY, E., SHIMIZU, K., GOLDFARB, M. & WIGLER, M. (1983). *Cell*, **34**, 581.

TAUB, R., KIRSCH, I., MORTON, C., LENOIR, G. SWARZ, D., TRONICK, S., AARONSON, S. & LEDER, P. (1982). Translocation of the c-*myc* gene into the immunoglobulin heavy chain locus in human Burkitt lymphoma and murine plasmacytoma cells. *Proc. Natl Acad. Sci.*, **79**, 7837.

TSICHLIS, P.N., STRAUSS, P.G. & HU, L.F. (1983). A common region for proviral DNA integration in MoMuLV-induced rat thymic lymphomas. *Nature*, **302**, 445.

WILLINGHAM, M.C., PASTAN, I., SHIH, T.Y. & SCOLNICK, E.M. (1980). Localization of the src gene product of the Harvey strain of MSV to the plasma membrane of transformed cells by electron microscopic immunocytochemistry. *Cell*, **19**, 1005.

YUASA, Y., SRIVASTAVA, S.K., DUNN, C.Y., RHIM, J.S., REDDY, E.P. & AARONSON, S.A. (1983). Acquisition of transforming properties by alternative point mutations with c-*bas/has* human proto-oncogene. *Nature*, **303**, 775.